# Drug-like analogues of the parasitic worm-derived immunomodulator ES-62 are therapeutic in the MRL/Lpr model of systemic lupus erythematosus

**DOI:** 10.1177/0961203315591031

**Published:** 2015-11

**Authors:** D T Rodgers, M A Pineda, C J Suckling, W Harnett, M M Harnett

**Affiliations:** 1 Institute of Infection, Immunity and Inflammation, Glasgow Biomedical Research Centre, University of Glasgow, Glasgow, UK; 2 Department of Pure and Applied Chemistry, University of Strathclyde, Glasgow, UK; 3 Strathclyde Institute of Pharmacy and Biomedical Sciences, University of Strathclyde, Glasgow, UK

**Keywords:** SLE, MRL/Lpr mouse, inflammation, antinuclear antibody (ANA), nephritis, parasitic helminth, ES-62, MyD88

## Abstract

**Introduction:**

ES-62, a phosphorylcholine (PC)-containing immunomodulator secreted by the parasitic worm *Acanthocheilonema viteae*, protects against nephritis in the MRL/Lpr mouse model of systemic lupus erythematosus (SLE). However, ES-62 is not suitable for development as a therapy and thus we have designed drug-like small molecule analogues (SMAs) based around its active PC-moiety. To provide proof of concept that ES-62-based SMAs exhibit therapeutic potential in SLE, we have investigated the capacity of two SMAs to protect against nephritis when administered to MRL/Lpr mice after onset of kidney damage.

**Methods:**

SMAs 11a and 12b were evaluated for their ability to suppress antinuclear antibody (ANA) generation and consequent kidney pathology in MRL/Lpr mice when administered after the onset of proteinuria.

**Results:**

SMAs 11a and 12b suppressed development of ANA and proteinuria. Protection reflected downregulation of MyD88 expression by kidney cells and this was associated with reduced production of IL-6, a cytokine that exhibits promise as a therapeutic target for this condition.

**Conclusions:**

SMAs 11a and 12b provide proof of principle that synthetic compounds based on the safe immunomodulatory mechanisms of parasitic worms can exhibit therapeutic potential as a novel class of drugs for SLE, a disease for which current therapies remain inadequate.

## Introduction

Systemic lupus erythematosus (SLE) is a chronic autoimmune disease in which inflammation and organ damage is driven by the deposition of antinuclear antibody (ANA)-containing immune complexes, with glomerulonephritis a major cause of morbidity. Despite recent advances in B cell-targeting therapies, there remains urgent need for safer, non-immunosuppressive therapies.^1^ Increasing epidemiological evidence that infection with parasitic helminths can protect against allergic and autoimmune inflammatory disorders has led to clinical trials that suggest that ‘worm therapy’ shows promise in the treatment of inflammatory conditions.^2^ Moreover, characterization of the actions of helminth-derived immunomodulators in preventing development of inflammatory disorders in experimental models has facilitated the identification of potential novel drug targets.^3^ Consistent with these advances, we have recently shown that prophylactic administration of ES-62, an immunomodulator secreted by the filarial nematode *Acanthocheilonema viteae,* can suppress ANA production and consequent kidney damage in the MRL/Lpr model of SLE^4^ by partially downregulating *myeloid differentiation factor 88* (MyD88) in B cells and kidney cells to reset the regulatory:effector B cell balance and desensitize renal effector function.^4^

However, the use of living pathogens as therapy is not ideal and to date, defined parasite-derived immunomodulators such as ES-62 have not been exploited as treatments due, for example, to their inherent problems of immunogenicity. We therefore recently designed a library of small drug-like compounds based around the active phosphorylcholine (PC)-moiety of ES-62, and presented proof of concept that these small molecule analogues (SMAs) provide novel prototypes for development of anti-inflammatory drugs for rheumatoid arthritis.^5^ We now report that two of these molecules, the sulfones 11a and 12b, protect against disease development when administered therapeutically after the onset of kidney damage, in the MRL/Lpr mouse and also by downregulating MyD88 levels, with consequent reduction of ANA and desensitization of kidney effector mechanisms.

## Methods

**Figure 1:**
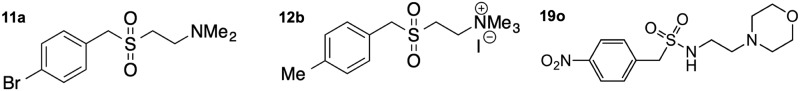
Structures of SMAs 11a, 12b and 19o. SMAs: small molecule analogues.

Female MRL/Lpr mice (Harlan-Olac) were maintained and assessed for proteinuria (Siemens Multistix analysis) in accordance with Home Office Licences PIL60/12183, PIL60/12950, PPL60/4492 and the ethics review board at the University of Glasgow.^4^ Endotoxin-free ES-62 and SMAs 11a, 12b and 19o (Figure 1) were prepared as described previously.^5^ Following development of proteinuria (>1 mg/ml), MRL/Lpr mice were treated twice-weekly with 11a, 12b or 19o (each at 1 µg in 100 µl phosphate-buffered saline (PBS)) or with PBS alone (100 µl), subcutaneously.

Kidneys were fixed and cryopreserved before being snap frozen in optimal cutting temperature (OCT) embedding medium, sectioned (7 µm), and stained with haematoxylin and eosin (H&E) or periodic acid-Schiff (PAS) before being visualized (×10 and ×40 magnification) using an Olympus BX41 camera. ANA were detected in mouse serum using human epithelial type 2 (HEp-2) slides (Antibodies Incorporated, USA) using a fluorescein isothiocyanate (FITC)-conjugated anti-mouse immunoglobulin (Ig)G antibody (Vector, USA) and visualized using an Axiovert fluorescent microscope at an original magnification of ×63. ANA levels in individual mice were determined by endpoint dilution analysis.^4^ Single kidney cell suspensions and supernatants were harvested and bone marrow-derived macrophages (bmM) differentiated in vitro before being analysed for cytokine production by enzyme-linked immunosorbent assay (ELISA) and MyD88 expression by flow cytometry and Western blotting (25 µg/sample; quantitative analysis by Image J software) as described previously.^4,5^ For flow cytometry, cells were fixed, permeabilized and stained for intracellular MyD88 using a rabbit anti-mouse-MyD88 antibody (Abcam) followed by an FITC-conjugated goat-anti-rabbit-antibody (Vector laboratories).^4,5^

Statistical analysis of proteinuria was by two-way analysis of variance (ANOVA) (Bonferroni’s post-test) whilst all other data were analysed by one-tailed Student’s *t*-test.

## Results

### Therapeutic administration of 11a and 12b suppresses proteinuria in the MRL/Lpr mouse

**Figure 2:**
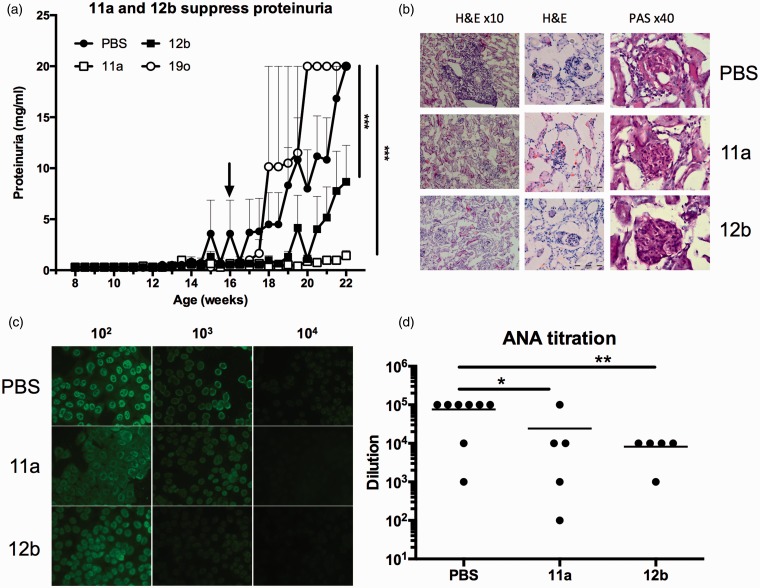
SMAs 11a and 12b, but not 19o, suppress proteinuria and ANA when administered therapeutically. (a) SMAs 11a, 12b, 19o, and PBS were administered following development of proteinuria (>1 mg/ml; arrow). Data presented are mean proteinuria scores ± SEM where *n* = 6 mice for all groups, except 19o where *n* = 2 and bars indicate range. (b) H&E and PAS staining of kidney sections from mice (after 22 weeks) treated with PBS, 11a or 12b at the indicated magnification with digital zoom images of glomeruli incorporating 100 µm scale bars. (c) Representative images of ANA staining of HEp-2 slides using the indicated serum dilutions from PBS-, 11a- or 12b-treated mice with ANA levels in individual mice shown in (d). SMAs: small molecule analogues; ANA: antinuclear antibodies; PBS: phosphate-buffered saline; HEp-2: human epithelial type 2; H&E: haematoxylin and eosin; PAS: periodic acid-Schiff.

A screen of drug-like SMAs based on the immunomodulatory PC-moiety of ES-62 identified 11a and 12b as mimicking the ability of the parasite product^5^ to suppress the Toll-like receptor (TLR)-mediated production of cytokines that promote the IL-17/IL-22 inflammatory axis which has been implicated in the pathogenesis of autoimmune diseases, including lupus.^4,5^ Thus, the therapeutic potential of 11a and 12b, relative to PBS and 19o, a structurally related inactive SMA,^5^ was assessed by twice-weekly administration (1 µg/dose) following onset of detectable proteinuria (>1 mg/ml; week 16) in female MRL/Lpr mice. Examination of the data generated revealed that treatment with 11a or 12b, but not 19o, suppressed proteinuria (Figure 2(a)). However, all of the 12b-treated mice developed some proteinuria (3 mg/ml; data not shown) and neither SMA prevented glomerular hypercellularity (Figure 2(b)). Nevertheless, protection against proteinuria was associated with reductions in cellular infiltration of the kidney and ANA production (Figure 2(b)–(d)).

### Mechanism of SMA action

**Figure 3:**
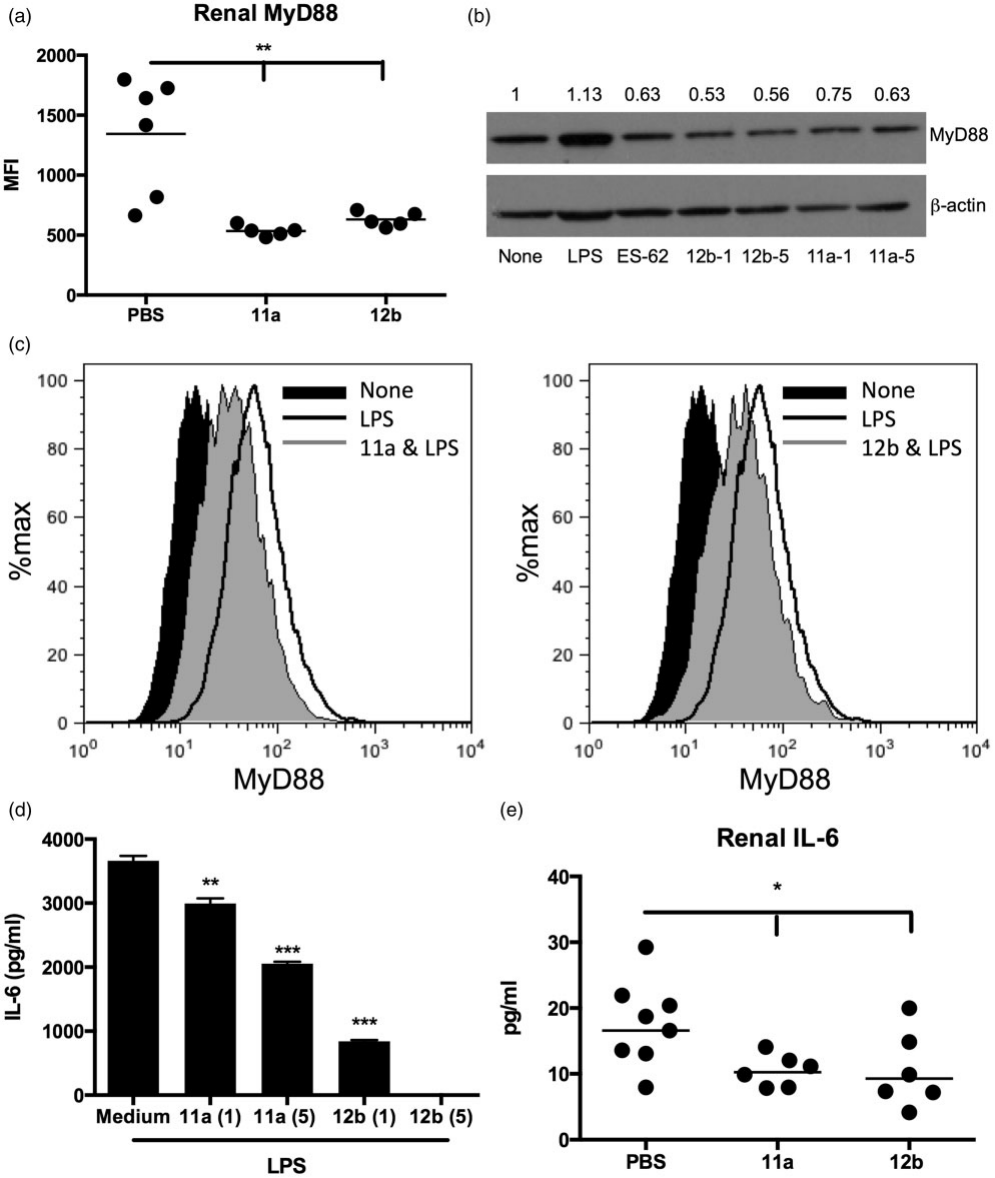
SMAs 11a and 12b suppress MyD88 signalling and downstream effector IL-6 responses in the kidney. (a) Flow cytometric analysis (MFI) of kidney MyD88 expression in individual mice (week 22). (b) MyD88 expression by bmM^5^ stimulated with LPS (1 µg/ml), ES-62 (2 µg/ml), 11a or 12b (both at 1 and 5 µg/ml) for 18 hours: exemplar Western blot and quantitation of MyD88/β-actin expression normalized to the ‘None’ (medium) control. (c) Flow cytometric analysis of MyD88 expression by bmM treated with 11a or 12b (5 µg/ml, two hours) prior to stimulation with LPS for 18 hours. IL-6 release by these cells (d), and in the kidney supernatants of PBS-, 11a- or 12b-treated (1 µg/dose) MRL/Lpr mice (e). SMAs: small molecule analogues; MFI: mean fluorescence intensity; LPS: lipopolysaccharide; IL-6: interleukin-6; PBS: phosphate-buffered saline; bmM: bone marrow-derived macrophages; MyD88:* myeloid differentiation factor 88*.

As with protection afforded by ES-62,^4^ both SMA 11a and 12b induced downregulation of MyD88 expression by kidney cells (Figure 3(a)). Moreover, when we analysed the levels of MyD88 in bmM derived from MRL/Lpr mice, as a surrogate for the inflammatory M1-like macrophages infiltrating the kidney during active lupus, it was found that in vitro treatment with 11a and 12b (1 and 5 µg/ml), but not 19o (results not shown), reduced the steady-state (to a comparable extent as ES-62; Figure 3(b)) and lipopolysaccharide (LPS)-upregulated levels of MyD88 in bmM (Figure 3(c)). Moreover, this 11a and 12b-mediated downregulation of MyD88 was associated with inhibition of LPS-mediated pro-inflammatory interleukin (IL)-6 responses (Figure 3(d)). Finally, analysis of the kidney supernatants^4^ of mice treated with 11a and 12b revealed that the protection afforded by the SMAs was associated with reduced levels of IL-6 (Figure 3(e)).

## Discussion

SMAs 11a and 12b suppress the development of proteinuria in the MRL/Lpr mouse, even after the onset of pathology, suggesting that SMAs such as these may have therapeutic potential in SLE. Indeed, the relevance of their therapeutic action is highlighted by reports that MyD88 signalling is essential for pathogenesis in the MRL/Lpr mouse,^6^ in particular by promoting the generation of interferon (IFN)α, IL-6 and ANAs required to drive autoimmune renal disease.^6,7^ Dissection of the pathogenic roles of MyD88 has indicated that deficiency solely in B cells is sufficient to protect against nephritis in both the MRL/Lpr and Lyn^–/–^ mouse models of lupus,^8,9^ as MyD88 signalling is essential for ANA production, activation of nephrotoxic T cells and glomerulonephritis. Reflecting this, ES-62 suppresses production of each of IL-6^+^ B cells, plasmablasts and ANA in MRL/Lpr mice and its desensitization of hyper-responsive B cells is associated with downregulation of their MyD88 expression and accompanied by an increased capacity of such B cells to express FcγRIIb, which mediates negative feedback regulation by immune complexes, and also produce the regulatory cytokine, IL-10.^4^ Indeed, the ability of splenic B cells from ES-62-treated MRL/Lpr mice to suppress lupus nephritis in recipient animals underlines the importance of downregulation of MyD88 expression in the ES-62-mediated resetting of the effector:regulatory B cell balance.^4^ Consistent with these findings in experimental models, MyD88 is required for the activation of antibody-producing plasma cells in patients^10^ and hence, together with its role in TLR/IL-1R signalling, has been identified as a therapeutic target in SLE.^11^ Moreover, given that ES-62 downregulates expression of MyD88 during IL-1-mediated induction of T helper (Th)17 cells^12^ and that there is evidence from studies on SLE patients that effector T cells are refractory to modulation by regulatory T cells (Tregs),^13^ recent findings suggesting that IL-1R-MyD88 signalling renders naive CD4^+^ T cells refractory to Tregs to allow Th1/Th17 differentiation^14^ provide an additional mechanism for suppressing lupus pathogenesis by targeting MyD88.

Like ES-62,^4^ SMA 11a and 12b act to maintain renal filter barrier and kidney functionality, as indicated by the significantly lower levels of proteinuria observed in treated mice, despite not inducing a global improvement in kidney histopathology. Rather, given its likely central role in integrating pathogenic crosstalk amongst TLR, complement and Fc receptors in SLE, this protection may be explained by the downregulation of MyD88 rendering renal cells hyporesponsive to such proinflammatory stimuli. This hypothesis is supported by the suppressed production of IL-6 by renal cells from MRL/Lpr mice exposed to 11a or 12b*,* the importance of which is reflected by the recent off-label success of IL-6-targeting biologics (anti-IL-6R monoclonal antibody (mAb), tocilizumab) in SLE patients, as well as the current phase II trials with anti-IL-6 mAbs in lupus nephritis (sirukumab) and in a randomized clinical trial (RCT) in SLE (PF-04236921).^1^ Moreover, whilst ES-62 selectively suppresses infiltration of the kidney by inflammatory T cells, neutrophils and M1-like macrophages, it increases the levels of tissue-protective M2-like macrophages that are deficient in lupus.^4^ Intriguingly, therefore, given that ES-62 and SMA 11a and 12b all downregulate MyD88 and IL-6 expression by kidney cells in vivo, and their inhibition of LPS-stimulated release of IL-6 by bmM derived from MRL/Lpr mice is associated with reduced MyD88 expression, it has recently been reported that deletion of MyD88 in endothelial and myeloid cells results in reduced recruitment of M1-like inflammatory macrophages, and abrogation of the pathogenic phenotypic switch from protective M2- to pathogenic M1-like macrophages in adipose tissue, in an obesity-related model of inflammatory disease.^15^

Perhaps surprisingly given their relative potencies in suppressing proteinuria in MRL/Lpr mice, SMA 12b was more effective than 11a in suppressing TLR4-mediated IL-6 production by bmM in vitro. However, it should be noted that the data obtained in vivo might be influenced by differences in the pharmacokinetics of the two SMAs. Nevertheless, our findings indicate SMA 11a to be the more effective at suppressing proteinuria. By resetting the level of MyD88 signalling in both infiltrating and resident kidney cells, SMAs such as 11a appear to act to suppress generation of pathogenic ANA and consequent immune complex-deposition and pro-inflammatory cell infiltration, responses which in concert drive IL-6-mediated lupus nephritis.^7^ Thus, such a drug-like compound, based on the safe, natural immunomodulatory action of ES-62 that has evolved over millennia to suppress aberrant, but not protective, inflammation, may provide the starting point for the development of novel therapies for SLE.

## References

[1] Stohl W Future prospects in biologic therapy for systemic lupus erythematosus. Nat Rev Rheumatol 2013; 9: 705–720. 24018550 10.1038/nrrheum.2013.136

[2] Weinstock JV Autoimmunity: The worm returns. Nature 2012; 491: 183–185. 23135449 10.1038/491183a PMC3744107

[3] Harnett W Harnett MM Helminth-derived immunomodulators: Can understanding the worm produce the pill? Nat Rev Immunol 2010; 10: 278–284. 20224568 10.1038/nri2730

[4] Rodgers DT McGrath MA Pineda MA The parasitic worm product ES-62 targets *myeloid differentiation factor 88*-dependent effector mechanisms to suppress antinuclear antibody production and proteinuria in MRL/Lpr mice. Arthritis Rheumatol 2015; 67: 1023–1035. 25546822 10.1002/art.39004 PMC4409857

[5] Al-Riyami L Pineda MA Rzepecka J Designing anti-inflammatory drugs from parasitic worms: A synthetic small molecule analogue of the *Acanthocheilonema viteae* product ES-62 prevents development of collagen-induced arthritis. J Med Chemi 2013; 56: 9982–10002. 10.1021/jm401251p PMC4125414 24228757

[6] Sadanaga A Nakashima H Akahoshi M Protection against autoimmune nephritis in MyD88-deficient MRL/Lpr mice. Arthritis Rheum 2007; 56: 1618–1628. 17469144 10.1002/art.22571

[7] Yung S Cheung KF Zhang Q Chan TM Mediators of inflammation and their effect on resident renal cells: Implications in lupus nephritis. Clin Dev Immunol 2013; 2013: 317682–317682. 24171032 10.1155/2013/317682 PMC3793320

[8] Teichmann LL Schenten D Medzhitov R Kashgarian M Shlomchik MJ Signals via the adaptor MyD88 in B cells and DCs make distinct and synergistic contributions to immune activation and tissue damage in lupus. Immunity 2013; 38: 528–540. 23499488 10.1016/j.immuni.2012.11.017 PMC3638041

[9] Hua Z Gross AJ Lamagna C Requirement for MyD88 signaling in B cells and dendritic cells for germinal center anti-nuclear antibody production in Lyn-deficient mice. J Immunol 2014; 192: 875–885. 24379120 10.4049/jimmunol.1300683 PMC4101002

[10] Capolunghi F Rosado MM Cascioli S Pharmacological inhibition of TLR9 activation blocks autoantibody production in human B cells from SLE patients. Rheumatology (Oxford) 2010; 49: 2281–2289. 20739362 10.1093/rheumatology/keq226

[11] Li J Wang X Zhang F Yin H Toll-like receptors as therapeutic targets for autoimmune connective tissue diseases. Pharmacol Ther 2013; 138: 441–451. 23531543 10.1016/j.pharmthera.2013.03.003 PMC3686650

[12] Pineda MA McGrath MA Smith PC The parasitic helminth product ES-62 suppresses pathogenesis in collagen-induced arthritis by targeting the interleukin-17-producing cellular network at multiple sites. Arthritis Rheum 2012; 64: 3168–3178. 22729944 10.1002/art.34581

[13] Buckner JH Mechanisms of impaired regulation by CD4(+)CD25(+)Foxp3(+) regulatory T cells in human autoimmune diseases. Nat Rev Immunol 2010; 10: 849–859. 21107346 10.1038/nri2889 PMC3046807

[14] Schenten D Nish SA Yu S Signaling through the adaptor molecule MyD88 in CD4+ T cells is required to overcome suppression by regulatory T cells. Immunity 2014; 40: 78–90. 24439266 10.1016/j.immuni.2013.10.023 PMC4445716

[15] Yu M Zhou H Zhao J MyD88-dependent interplay between myeloid and endothelial cells in the initiation and progression of obesity-associated inflammatory diseases. J Exp Med 2014; 211: 887–907. 24752299 10.1084/jem.20131314 PMC4010914

